# Predicting the Growth of *Vibrio parahaemolyticus* in Oysters under Varying Ambient Temperature

**DOI:** 10.3390/microorganisms11051169

**Published:** 2023-04-29

**Authors:** Iker Fernández-Vélez, Gorka Bidegain, Tal Ben-Horin

**Affiliations:** 1 Department of Preventive Medicine and Public Health, University of the Basque Country (UPV/EHU), Barrio Sarriena s/n, 48490 Leioa, Spain; 2 Department of Applied Mathematics, Engineering School of Bilbao, University of the Basque Country (UPV/EHU), Plaza Ingeniero Torres Quevedo 1, 48013 Bilbao, Spain; 3 Research Centre for Experimental Marine Biology & Biotechnology, Plentzia Marine Station, University of the Basque Country (PiE-UPV/EHU), Areatza Pasealekua, 48620 Plentzia, Spain; 4 College of Veterinary Medicine, North Carolina State University, 303 College Circle, Morehead City, NC 28557, USA

**Keywords:** *Vibrio*, oysters, post-harvest, modeling, temperature, ice treatment

## Abstract

Temperature is a critical factor that influences the proliferation of pathogens in hosts. One example of this is the human pathogen *Vibrio parahaemolyticus* (*V. parahaemolyticus*) in oysters. Here, a continuous time model was developed for predicting the growth of *Vibrio parahaemolyticus* in oysters under varying ambient temperature. The model was fit and evaluated against data from previous experiments. Once evaluated, the *V. parahaemolyticus* dynamics in oysters were estimated at different post-harvest varying temperature scenarios affected by water and air temperature and different ice treatment timing. The model performed adequately under varying temperature, reflecting that (i) increasing temperature, particularly in hot summers, favors a rapid *V. parahaemolyticus* growth in oysters, resulting in a very high risk of gastroenteritis in humans after consumption of a serving of raw oysters, (ii) pathogen inactivation due to day/night oscillations and, more evidently, due to ice treatments, and (iii) ice treatment is much more effective, limiting the risk of illness when applied immediately onboard compared to dockside. The model resulted in being a promising tool for improving the understanding of the *V. parahaemolyticus*–oyster system and supporting studies on the public health impact of pathogenic *V. parahaemolyticus* associated with raw oyster consumption. Although robust validation of the model predictions is needed, the initial results and evaluation showed the potential of the model to be easily modified to match similar systems where the temperature is a critical factor shaping the proliferation of pathogens in hosts.

## 1. Introduction

Oysters, traditionally harvested for thousands of years, are a very important part of many diets around the world [1] and increasing as aquaculture oyster demands surge [2]. Since 2009, global production has increased from 3 million to over 5.9 million tons [3]. This is an important factor contributing to the increase in the number of cases of gastroenteritis caused by the human pathogen *Vibrio parahaemolyticus* (*V. parahaemolyticus*) [4,5], a bacteria endemic to marine environments and present in seafood, including oysters [6]. Among bivalves, the oyster is the one most-involved in outbreaks because it is traditionally eaten raw [7,8]. In addition to gastroenteritis, consuming raw oysters with high *V. parahaemolyticus* concentrations can lead to primary septicemia in individuals with underlying medical conditions such as chronic diseases, liver disease, or immune disorders [9].

The first outbreak of *V. parahaemolyticus* was reported in Osaka (Japan) in 1950, followed by a continuing rise of *V. parahaemolyticus* infections in several countries over the past few decades [10,11,12,13]. In 2020, around half a million *V. parahaemolyticus* infection cases were estimated worldwide [12]. Thus, this pathogen is the leading cause of seafood-related bacterial illness worldwide [14]. However, it has to be noted that not all *V. parahaemolyticus* strains are pathogenic [15].

It is almost impossible to obtain seafood free of these bacteria due to the ubiquitous nature of *Vibrio* species in marine and estuarine environments, particularly during warm, summer months [8,16,17], and the accumulation of *Vibrio* by oysters through filter-feeding. Seawater temperature is a major factor determining the concentration, distribution, and proliferation of *V. parahaemolyticus* in the coastal environment [16,18]. Higher densities of *V. parahaemolyticus* in oysters have been detected in the spring and summer and are positively correlated with seawater temperature [16,19]. *V. parahaemolyticus* is rarely detected during winter, when *Vibrio* survives in the marine sediment until temperatures rise again to 14 °C and it is released to the seawater [2]. Oysters harvested in summer can be associated with *V. parahaemolyticus* tissue concentrations approaching 1000 CFU/g, while in winter, this concentration decreases to less than 10 CFU/g [16,20].

Oysters are harvested, sacked, and left at ambient air temperature on the boat deck before being dragged to shore. When the air temperature is very high in hot summer conditions, this exposure can result in an important *V. parahaemolyticus* growth in oysters up to $50\times 10^{6}$ cells/g (7.7 $log_{10}$ CFU/g) [21]. Regarding disease risk, the probability of illness is relatively low in winter (≤0.00001) for consumption of a serving of 12 oysters with $100\times 10^{3}$ *V. parahaemolyticus* cells (∼50 cells/g or 1.7 $log_{10}$ CFU/g) [22]. However, in summer, this probability can increase to 0.5 after the consumption of a serving with $100\times 10^{6}$ *V. parahaemolyticus* cells (∼ $500\times 10^{3}$ cells/g or 5.7 $log_{10}$ CFU/g) [22]. In this context, the rising temperature associated with climate change is a factor of concern, due to the expected influence of prolonged exposure to seawater temperatures supporting *Vibrio* proliferation and its impact on *V. parahaemolyticus* distribution [12,13] and population dynamics, and eventually the impact on human disease outcomes.

To address this risk, worldwide sanitation programs for shellfish control established time-to-temperature regulations to limit the growth of *V. parahaemolyticus* in post-harvest oysters [23]. An example of this thermal process consists of cooling down the shellfish harvested for raw consumption to 10 °C (50 °F) within 10, 12, or 36 h when the average monthly air temperature is higher than 27 °C, between 19 and 27 °C, respectively [2]. An absence of refrigeration, non-rapid refrigeration, or breaking the cold chain can lead to high temperatures during oyster warehousing, leading to the *V. parahaemolyticus* in vivo population increase. Resubmersion can also be considered a method for *Vibrio* control [24].

This effect of temperature on *V. parahaemolyticus* has been commonly explored by experiments at different constant temperatures [21,25,26]. However, inferring outcomes from constant-temperature experiments for realistic varying temperature regimes is complex and problematic [27]. Improving methods in this regard is essential for the understanding of this and similar temperature-dependent host–pathogen systems while supporting studies about the public health impact of pathogenic *V. parahaemolyticus* associated with raw seafood consumption [23]. Predictive models addressing this for the *V. parahaemolyticus*–oyster system are scarce. Fernández-Piquer et al. [28] developed a preliminary stochastic model at constant temperatures and generated probabilistic distributions and predictions for the percentage of oysters containing high levels of *V. parahaemolyticus* for each simulated temperature scenario. Ndraha and Hsiao [17] assessed the risk of *V. parahaemolyticus* in raw oysters in Taiwan for different seasons and climate scenarios. Love et al. [29] formulated a simple iterative temperature-based tow equation model using a temperature threshold of 5.4 °C for estimating bacterial growth/die-off when exposed to temperatures above/below this refrigeration threshold. For this model, only bacterial growth was temperature-dependent, and the simulation results were not evaluated against real *V. parahaemolyticus* data.

In this study, a continuous-time model was developed for predicting the growth of *V. parahaemolyticus* in oysters under varying ambient temperatures. Thus, this model is one step forward for modeling this system: it was constructed by numerically integrating an ordinary differential equation system with temperature-dependent growth and inactivation parameters and a temperature threshold for growth/inactivation of *V. parahaemolyticus* adapted from [21]. First, the model was fit, verified, and evaluated against previous experimental data of *V. parahaemolyticus* concentrations in Pacific oysters (*Crassostrea gigas*) at constant temperatures [21]. Once the model was verified and evaluated, the *V. parahaemolyticus* dynamics in oysters were modeled for different post-harvest scenarios under varying environmental temperatures for winter and summer initial conditions and with and without dockside ice and onboard ice treatments.

## 2. Materials and Methods

The predictive model developed in this study for exploring *V. parahaemolyticus* growth in oysters under varying environmental temperatures was parameterized with and evaluated against experimental data of *V. parahaemolyticus* concentrations ($log_{10}$ CFU/g) in Pacific oysters obtained at different constant temperatures (see the details in [21]). First, linear and non-parametric regression models were obtained. Second, from the maximum slopes of these models, the inactivation and growth rates were estimated. Third, using these rates, our growth model was constructed for predicting the growth of *V. parahaemolyticus* in oysters under varying ambient temperatures:

### 2.1. OLS and LOESS Regression Models for *V. parahaemolyticus* Growth and Inactivation Processes

Two models were applied to analyze the relationship of *V. parahaemolyticus* concentration in oysters with time at experimental constant temperatures [21]: the classic parametric Ordinary Least-Squares regression model (OLS) [30] and the non-parametric Locally Weighted Least-Squares Regression smoothing technique (LOESS) [31]. As a nonparametric smoothing technique, the LOESS can model the relationship between *Vp* and time more robustly and in a more flexible manner than parametric models such as the OLS, potentially extracting information (e.g., ecological, biological) from the data that more restrictive parametric models miss. For *V. parahaemolyticus* inactivation, the goodness of fit of the OLS and LOESS regression curves were assessed and compared by analyzing the Percentage Error (PE); a lower PE means a better prediction accuracy. The PE was calculated based on the Residual Standard Error (RSE) as follows:

(1) $$\begin{array}{c} RSE=\sqrt{\frac{\sum_{i=1}^{n}\left(VPC_{i}-\hat{VPC_{i}}\right)}{n}} \end{array}$$

(2) $$\begin{array}{c} PE\left(\%\right)=100\;\cdot \;\frac{RSE}{\bar{VPC}} \end{array}$$

where $VPC_{i}$ and $\hat{VPC}$ are, respectively, the observed *V. parahaemolyticus* and mean *V. parahaemolyticus* counts in terms of colony-forming units ($log_{10}$ CFU/g) and $\hat{VPC_{i}}$ are the predicted counts by the OLS and LOESS models.

For *V. parahaemolyticus* growth, the goodness of fit of the LOESS regression curve was assessed. The obtained curves and the corresponding maximum slopes or growth rates were compared to the curves obtained by model fitting in [21]. The PE was also calculated here based on the residual standard error.

Data analysis was conducted with the R statistical software (Version 4.3.0) (R Core Team, 2018). The “ggplot2” package was used to generate the plots.

### 2.2. Growth and Inactivation Rates for the Model

For *V. parahaemolyticus* growth, the classic square root model [32] was applied to describe the growth rate (*r*) as a function of temperature as follows:

(3) $$\begin{array}{c} \sqrt{r}=a\;\cdot \;\left(T-T_{min}\right). \end{array}$$

The model shows a linear relationship between temperature (*T*) and the $\sqrt{r}$, where the regression coefficient is represented by a and $T_{min}$ is a hypothetical reference or threshold temperature between the growth and inactivation of *V. parahaemolyticus*.

The Arrhenius equation was used for the estimation of the kinetic parameters for the effect of temperature on bacterial inactivation as follows:

(4) $$\begin{array}{c} \ln r=\ln A-\frac{E_{a}}{R\;\cdot \;T} \end{array}$$

where *r* is the reaction rate coefficient or constant, *T* is the absolute temperature, $E_{a}$ is the activation energy (i.e., the minimum amount of energy that must be provided to result in a reaction), *R* is the universal gas constant, and *A* is the collision factor.

### 2.3. The Model

#### 2.3.1. Model Description, Mathematical Theory, and Assumptions

The model developed here is a *V. parahaemolyticus* growth model for *V. parahaemolyticus* in oysters that accounts for the effect of varying temperature on bacterial growth. It is a continuous-time model, which results from an Ordinary Differential Equation (ODE) system solved using a Fourth-order Runge–Kutta method (RK4) [33]. The numerical model for this ODE system was programmed in Matlab.

The model accounts for both bacterial growth and inactivation. Regarding bacterial growth, the model is an extension of the logistic model [34], which suggests that the rate of the bacterial population increase is limited. The logistic model combines the ecological processes of growth and competition. Both processes depend on population density, and their rates match the mass–action law [34]. Regarding bacterial inactivation, the model is a linear decreasing model.

#### 2.3.2. Model Equations

*V. parahaemolyticus* growth and inactivation are described by the system and conditions defined by Equations (5) and (6), $N=N_{1}+N_{2}$ being the total number of *V. parahaemolyticus* counts per gram. That is, to have both *V. parahaemolyticus* growth and inactivation in the equation system for the variable, the *V. parahaemolyticus* population in oysters was divided into two classes, $N_{1}$ and $N_{2}$. The following equations represent the change of these two classes with time:

(5) $$\begin{array}{c} \frac{d\;N_{1}}{d\;t}=-\gamma \;\mu_{max}\;N_{1} \end{array}$$

(6) $$\begin{array}{c} \frac{d\;N_{2}}{d\;t}=\gamma \;\mu_{max}\;m\;N_{1}\;+\;\mu_{max}\;N_{2}\left(1-\frac{m\;(N_{1}\;+\;N_{2})}{N_{max}}\right), \end{array}$$

where $\mu_{max}$ is the maximum growth rate of the *V. parahaemolyticus* population in oysters (cells/h). $N_{max}$ accounts for substrate competition, that is it represents the carrying capacity or the maximum total counts, corresponding to 5.5 $log_{10}$ CFU/g for lower temperatures (*T* < 23 °C), 7.5 $log_{10}$ CFU/g for medium-high temperatures (23 °C ≤ *T* < 30 °C), and 6.75 $log_{10}$ CFU/g for high temperatures (*T* ≥ 30 °C), as observed by [21]. The parameter *m* can be 1 or 0, depending on the temperature (see Equations (7) and (8)).

From Equations (3) and (4), the following growth rates ($\mu_{max}$) were obtained:

(7) $$\mathrm{if}\;T\;>\;T_{min},\;\mu_{max}\;=\;a^{2}\left(T-T_{min}\right)\;\mathrm{and}\;m\;=\;1$$

(8) $$\mathrm{if}\;T\;\leq \;T_{min},\;\mu_{max}\;=\;e^{\ln A-\frac{E_{a}}{R\;\cdot \;T}}\;\mathrm{and}\;m\;=\;0$$

The growth rate $\mu_{max}$ and *m* depend on the temperature (*T*), representing the growth or inactivation of *V. parahaemolyticus* in oysters. That is, when *T* is higher than 13.37 °C, the *V. parahaemolyticus* population growth is defined by Equation (7), being *m* = 1, so that the first term in both Equations (5) and (6) is annulled. In these conditions, the model studies the change in the total *V. parahaemolyticus* population $N=N_{1}+N_{2}$ or bacterial growth by the second term in Equation (6), $\mu_{max}\;N_{2}\left(1-\frac{m\;(N_{1}\;+\;N_{2})}{N_{max}}\right)$. If *T* is lower than or equal to 13.37 °C, the growth rate $\mu_{max}$ is defined by Equation (8), being *m* = 0. In these conditions, the change in the total *V. parahaemolyticus* population ($N=N_{1}+N_{2}$ or inactivation is only defined by $-\gamma \;\mu_{max}\;N_{1}$ in Equation (5). The rest of the terms are annulled when *m* = 0 and the initial number of $N_{2}=0$.

#### 2.3.3. Model Verification and Evaluation

The probability of illness is relatively low (<0.001 percent) for the consumption of $10\times 10^{3}$ cells/g *V. parahaemolyticus* cells/serving [22], a serving being 12 oysters or approximately 16 g of meat. This is equivalent to about 50 cells/oyster meat gram, that is 800 cells/serving. These concentrations are equivalent to the winter CFU/g values [16]. However, the probability of disease increases to 50 percent for consumption of about $100\times 10^{6}$ *Vp* cells/serving. This corresponds to $8000\times 10^{3}$ cells/oyster [22], which are body burdens of the same order of magnitude as those found in oysters harvested in summer [16].

Model verification consisted of showing that the model is correct, complete, and coherent by means of (i) static tests involving a structured examination of the formulas, algorithms, and code used to implement the model and (ii) dynamic tests, where the computer program was run under different conditions to ensure that the results produced were correct, according to the conceptual model, and consistent with the expectations of the reviewer experts in oyster pathology, *V. parahaemolyticus*, and population dynamics.

The parameter values used for model verification were those obtained by fitting the model growth and inactivation rate equations (Equations (5) and (6)). The model was run for a series of 100/150 h of simulation for growth scenarios and 300/500 h of simulations for inactivation scenarios. This simulation time span was chosen (i) to detect pathogen proliferation and inactivation events and (ii) to evaluate the model against growth and inactivation experimental data along the same time span [21]. Thus, eight realistic (experimental) scenarios were simulated to verify and evaluate the performance of the model regarding the dynamics of the *V. parahaemolyticus* population (Table 1) [21].

#### 2.3.4. Modeling Scenarios

Different simulations were run representing both (i) realistic environmental (water and air) temperatures for regions with hot summers and mild winters, as in Southern Europe [35] and the Southern U.S. [36], and (ii) realistic oyster processing temperature scenarios in terms of refrigeration by ice treatment from harvesting to consumption, in both summer and winter (Simulations 9–17). For each season, the simulated scenarios were differentiated by the ice treatment applied to oysters: Non-Iced (NI), Dockside Ice Storage (DS), and Onboard ice storage (OB). In the NI scenario, there is no pre-consumption treatment in terms of refrigeration. In the DS scenario, ice treatment starts 10 h after harvesting. In the OB scenario, ice treatment starts onboard right after harvesting.

Four other realistic scenarios of interest were run in order to explore how *V. parahaemolyticus* growth would behave in the event of a potential break in the cold chain (Simulations 18–21) referring to dockside and onboard situations in both winter and summer. The last simulation represents the *V. parahaemolyticus* inactivation scenario in the long term. The characteristics of this set of modeling scenarios are summarized in Table 2.

### 2.4. Risk of Illness

The model results are discussed in terms of the probability of illness. For this, the risk of illness was estimated in Table 3 by adapting previous dose–response model results [22].

Note that U.S. Food and Drug Administration [22], for example, predicts about 2800 *V. parahaemolyticus* illnesses from oyster consumption each year. Of infected individuals, approximately 7 cases of gastroenteritis will progress to septicemia each year for the total population, of which 2 individuals would be from the healthy sub-population and 5 would be from the immunocompromised sub-population [22].

### 2.5. Model Limitations

The ODE system here was solved using the RK4 method. RK4 methods are easy to implement, very stable, and self-starting; that is, unlike multi-step methods, there is no need to treat the first few steps taken by a single-step integration method as special cases. However, the primary disadvantages of RK4 methods are (i) the requirement of significantly more computation time than multi-step methods of comparable accuracy and (ii) the fact that they do not easily yield good global estimates of the truncation error. However, for straightforward dynamical systems such as the one investigated by this model, the advantage of the relative simplicity and ease of use of RK4 methods far outweighs the disadvantage of their relatively high computational cost.

The growth and inactivation rate for the model was obtained by integrating the information obtained by regression models exploring the change in *V. parahaemolyticus* concentrations with time at only eight constant temperatures. This is a simplification of reality and may result in a relative underestimation of *V. parahaemolyticus* growth and, consequently, is an underestimation of the risk gastroenteritis and septicemia.

Finally, when modeling realistic scenarios, caution is required to interpret the results in quantitative terms, since the model deals with multiple dimensions, latent covariates, and data coming from laboratory experiments, which could result in some situations that are not entirely realistic.

## 3. Results

### 3.1. OLS and LOESS Regression Models for V. parahaemolyticus

**Figure 1:**
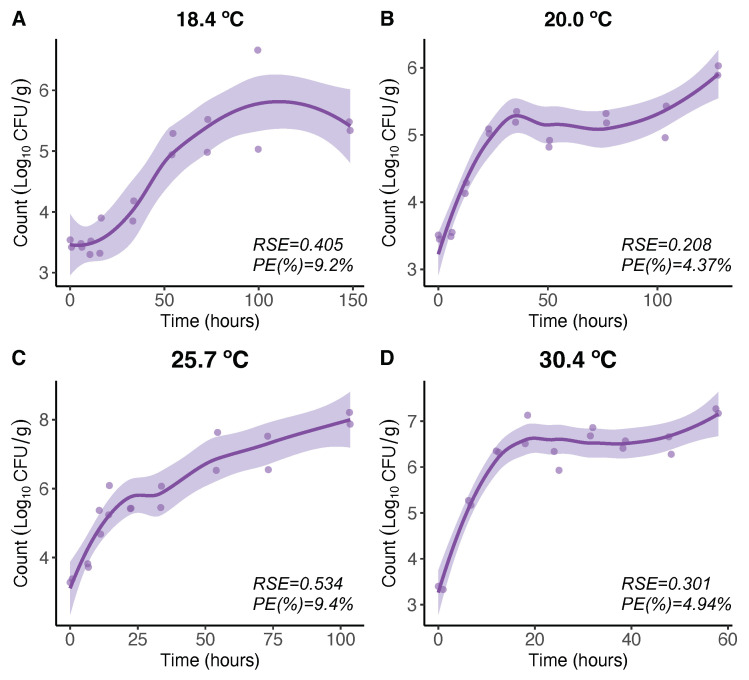
LOESS regression model for 18.4 (**A**), 20.0 (**B**), 25.7 (**C**), and 30.4 °C (**D**). Regression curves are represented by solid lines with smoothing based on 95% confidence intervals (shaded areas).

For *V. parahaemolyticus* growth, at higher temperatures (18.4 °C, 20.0 °C, 25.7 °C, and 30.4 °C), the LOESS model expresses the *V. parahaemolyticus* growth in a more flexible manner than that obtained by the Baranyi model [21]. The maximum *V. parahaemolyticus* count level beyond which the *V. parahaemolyticus* counts remain constant in the Baranyi model [21] is not that clear and constant for the LOESS model here, showing some increasing patterns beyond that level (Figure 1A–D). Nevertheless, comparing the maximum growth rates (initial slopes) there were no differences.

**Figure 2:**
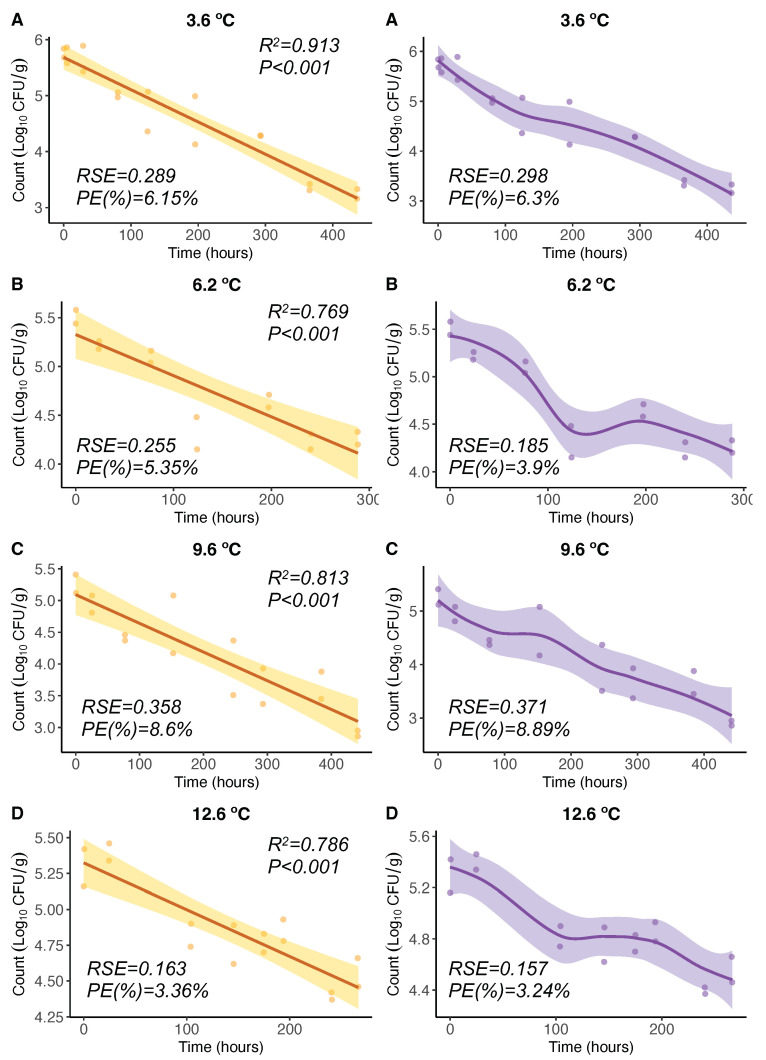
OLS (**left**) and LOESS (**right**) regression models for 3.6 (**A**), 6.2 (**B**), 9.6 (**C**), and 12.6 °C (**D**). Regression curves are represented by solid lines with smoothing based on 95% confidence intervals (shaded areas).

For *V. parahaemolyticus* inactivation, OLS regression models from experiments [21] showed significant (*p* < 0.05) negative linear relationships of *V. parahaemolyticus* counts (CFU/g) with time for different constant temperatures between 3.6 °C and 12.6 °C (Figure 2A–D, left). The Pearson correlation coefficient squared was high in all cases ($R^{2}$ between 0.769 and 0.913). The LOESS regression curves showed that the response variable *V. parahaemolyticus* counts exhibited a progressive inactivation (decrease) with time (Figure 2A–D, right). In particular, for 6.2 °C and 12.6 °C, this inactivation showed two phases: the inactivation in the first phase was faster than that in the second phase. These LOESS curves adapted in a more flexible manner to the data; however, the differences in the PE were not significant, and inactivation rates can be considered similar. The OLS and LOESS showed similar PE, 3.36–8.6% and 3.24–8.89%, respectively. Regarding the goodness of fit, all studied models were well-fitting regression models (PE < 10%).

The results of this comparative analysis between both the OLS and Baranyi models applied in [21] and the LOESS models here suggested using similar maximum growth rate values (slopes) for each temperature as in [21] in order to formulate the equations (models) for the growth and inactivation rates needed for the *V. parahaemolyticus* growth model in oysters developed here.

### 3.2. Growth and Inactivation Rates for the Model

From OLS and LOESS regression models (slopes), maximum growth and inactivation rates for *V. parahaemolyticus* were obtained as in [21]. These rates were −0.006, −0.004, −0.005, −0.003, 0.030, 0.075, 0.095, and 0.282 $log_{10}$ CFU/h at 3.6, 6.2, 9.6, 12.6, 18.4, 20.0, 25.7, and 30.4 °C, respectively. Fitting these rates vs. different temperatures, the following parameter values for Equations (1) and (2) were obtained: *T*_min_ = 13.37 °C, as an intrinsic property of the Pacific oyster, and a=0.04, $A=4.81\times 10^{-9}$, $E_{a}=4131.2$, R=T+273.5. Thus, the growth and inactivation rate models as a function of temperature were formulated as follows:

For *V. parahaemolyticus* growth, the classic square root model [32] applied to describe the growth rate is formulated as:

(9) $$\begin{array}{c} \sqrt{r}=0.04\;\cdot \;\left(T-13.37\right). \end{array}$$

The model for growth shows a linear relationship between temperature (*T*) and the $\sqrt{r}$, where the regression coefficient is represented by *a* and $T_{min}$ is a hypothetical reference or threshold temperature between the growth and inactivation of *V. parahaemolyticus*. As an intrinsic property of the Pacific oyster, this temperature is *T*_min_ = 13.37 °C [21].

The Arrhenius equation was used for the estimation of the kinetic parameters for the effect of temperature on bacterial inactivation. The equation used is as follows:

(10) $$\ln r=Ln(4.81\;\cdot \;10^{-9}+4131.2\;\cdot \;\left(\frac{1}{T+273.5}\right)),$$

where *r* is the rate constant, *T* is the absolute temperature, $E_{a}$ is the activation energy, *R* is the universal gas constant, and *A* is the collision factor.

### 3.3. Model Verification and Evaluation

**Figure 3:**
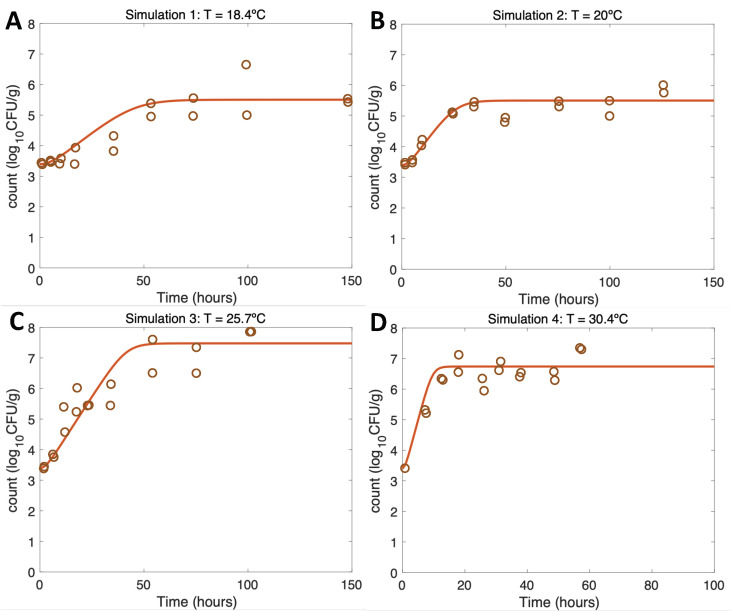
Simulations 1–4 from Table 1 (**A**–**D**) for model verification and evaluation using constant temperatures as in [21].

**Figure 4:**
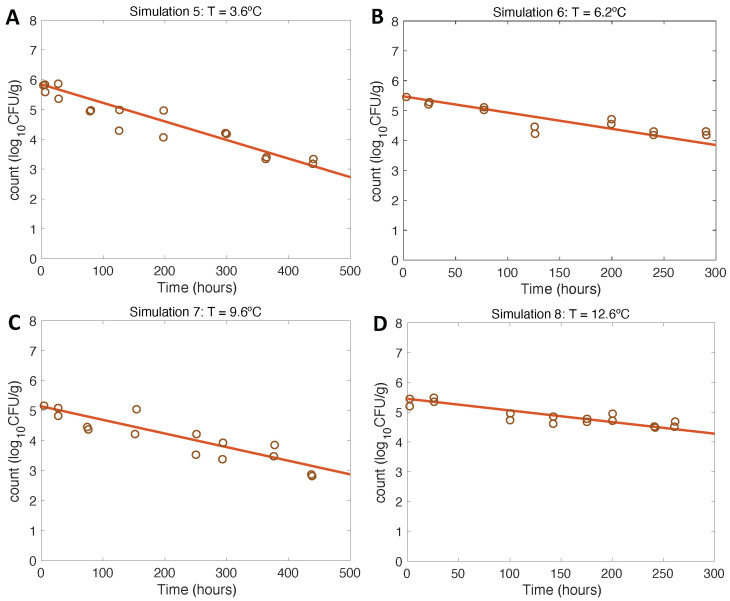
Simulations 5–8 from Table 1 (**A**–**D**) for model verification and evaluation using constant temperatures as in inactivation experiments conducted by [21].

Simulations at constant temperatures for model verification and evaluation (Figure 3 and Figure 4) were run with the initial conditions described in Table 1 and the model parameter values defined in Section 2.3.3 and Section 3.2.

For growth scenarios (Simulations 1–4), the results conformed to the expectations (Table 1) and, thus, were consistent with the real experimental data from [21] using identical initial conditions and temperatures. Increasing the temperature from Figure 3A–C led to more rapid initial growth and eventually to a higher maximum growth rate, conforming to the expectations (Table 1). Overall, regarding the model evaluation, the model had a very good fit to the experimental data both for the maximum growth and the *V. parahaemolyticus* values at the maximum of the curve (Table 1 and [21]).

The results of model verification for inactivation scenarios conformed to the expectations, being also consistent with the experimental data from [21]. In this case, the opposite trend to growth was observed: the lower the temperature, the faster the inactivation of the pathogen conformed to the expected final values. Regarding model evaluation, the model had a very good fit to the experimental data for the entire inactivation pattern (Table 1 and [21]).

### 3.4. Modeling Scenarios under Varying Temperature

**Figure 5:**
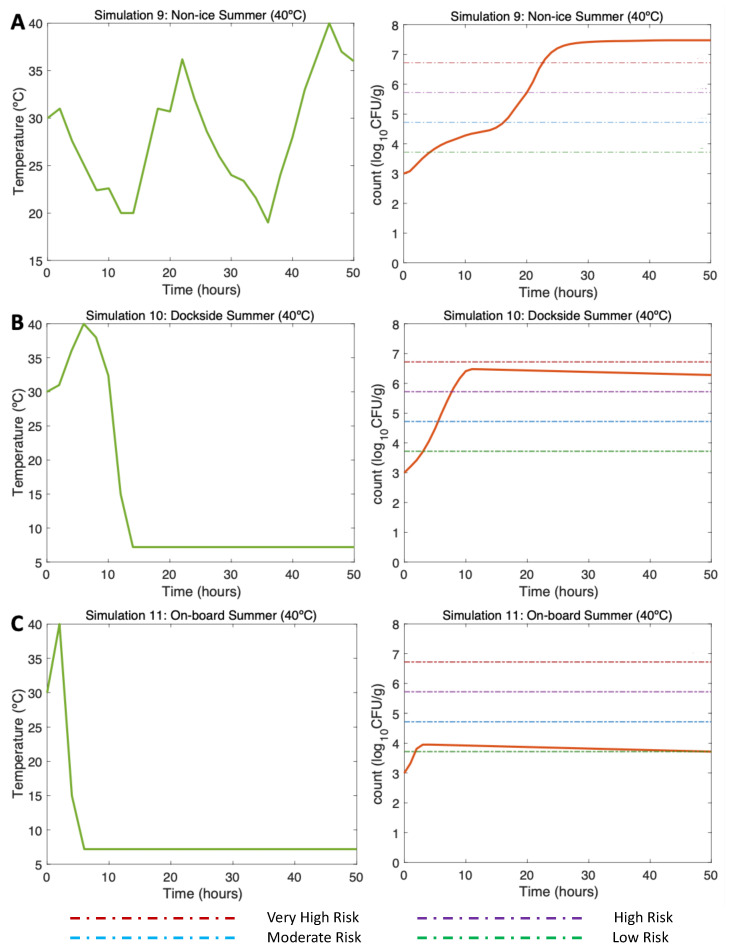
Simulations 9–11 from Table 2. Summer, (**A**) Non-Ice (NI), (**B**) Dockside (DS), and (**C**) Onboard (OB) post-harvest ice treatment scenarios. Water temperature of 30 °C, air temperature of 40 °C, and initial *V. parahaemolyticus* concentration of 1000 CFU/g.

**Figure 6:**
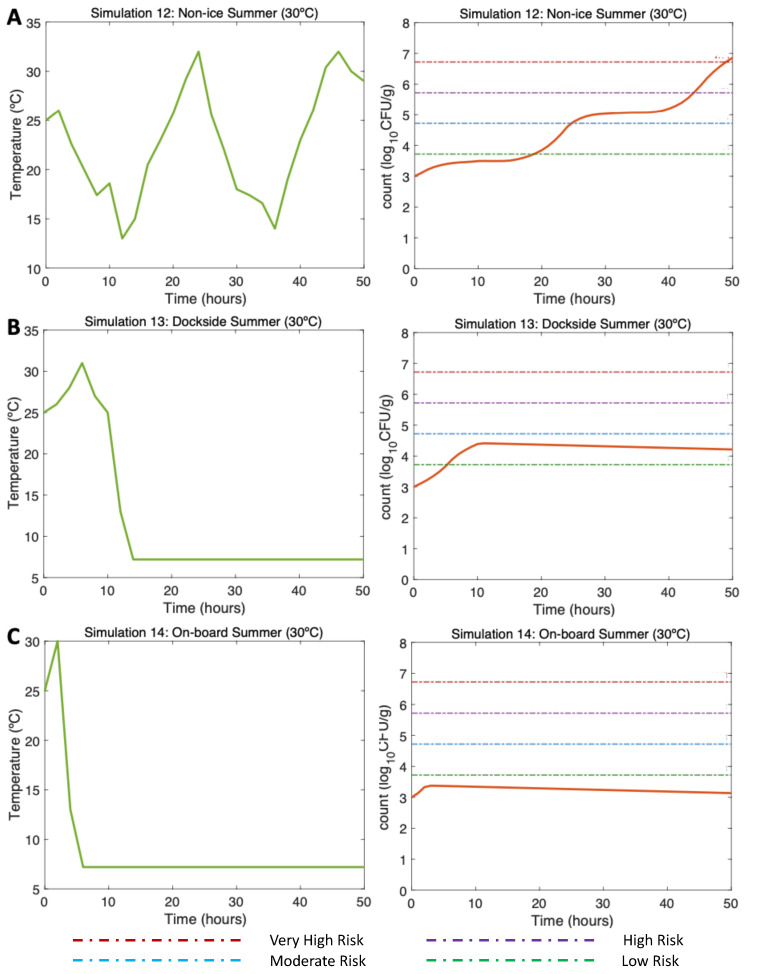
Simulations 12–14 from Table 2. Summer, (**A**) Non-Ice (NI), (**B**) Dockside (DS), and (**C**) Onboard (OB) post-harvest ice treatment scenarios. Water temperature of 25 °C, air temperature of 30 °C, and initial *V. parahaemolyticus* concentration of 1000 CFU/g.

**Figure 7:**
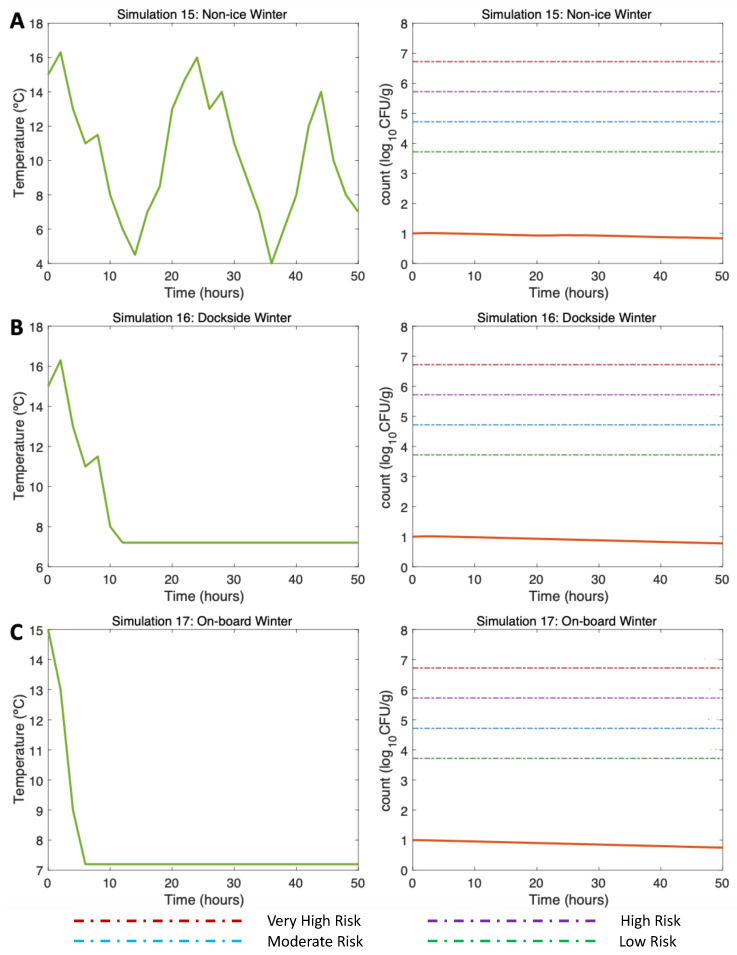
Simulations 15–17 from Table 2. Winter, (**A**) Non-Ice (NI), (**B**) Dockside (DS), and (**C**) Onboard (OB) post-harvest ice treatment scenarios. Water temperature of 15 °C and initial *Vp* concentration of 10 CFU/g.

**Figure 8:**
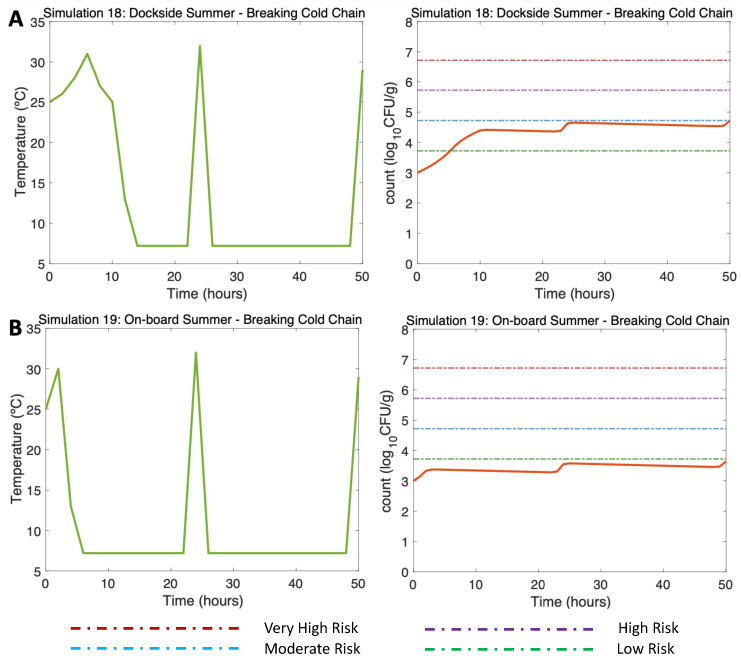
Simulations 18–19 from Table 2. Summer, (**A**) Dockside (DS) and (**B**) Onboard (OB) post-harvest ice treatment scenarios, breaking the cold chain. Water temperature of 25 °C, a maximum air temperature of 32 °C, and initial *Vp* concentration of 1000 CFU/g.

**Figure 9:**
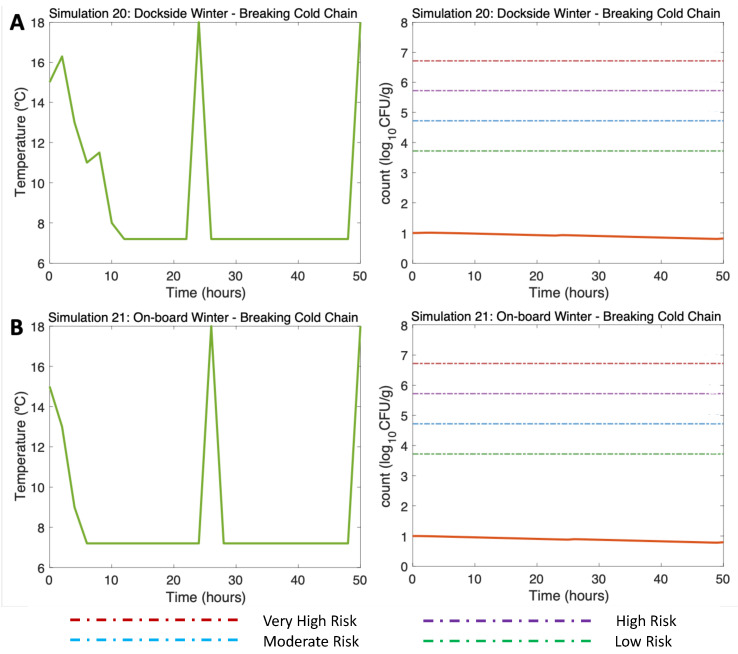
Simulations 20–21 from Table 2. Winter, (**A**) Dockside (DS) and (**B**) Onboard (OB) post-harvest ice treatment scenarios, breaking the cold chain. Water temperature of 15 °C, a maximum air temperature of 18 °C, and initial *V. parahaemolyticus* concentration of 10 CFU/g.

**Figure 10:**
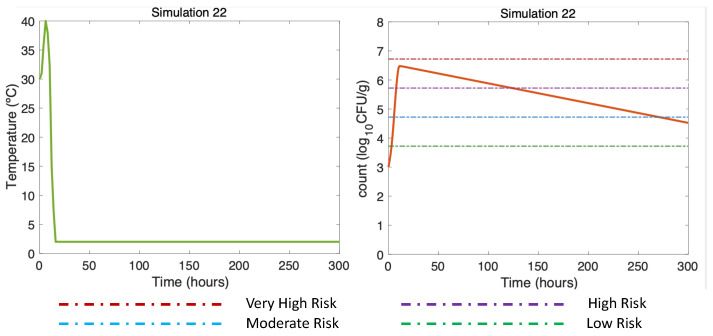
Summer Dockside (DS) post-harvest ice treatment scenario, 300 h duration (Table 2). Water temperature of 30 °C, maximum air temperature of Air 40 °C, and initial *V. parahaemolyticus* concentration of 1000 CFU/g.

Once the model was verified and evaluated against real data [21], a series of theoretical scenarios was simulated, trying to mirror realistic scenarios. The results obtained for each scenario tested are described and shown in Table 2 and Figure 5, Figure 6, Figure 7, Figure 8, Figure 9 and Figure 10.

#### 3.4.1. Simulations 9–11: Summer, Water 30 °C, Air (Max) 40 °C

In the NI scenario (Simulation 9; Figure 5A, left), the initial *V. parahaemolyticus* concentration was 1000 CFU/g (3 $log_{10}$ CFU/g), as found by [16] in summer. The post-harvest air temperature oscillated between 18 and 40 °C, mirroring day/night temperature fluctuations. Given these varying temperature conditions, a gradual increase in the *V. parahaemolyticus* counts was observed with a maximum of 7.5 $log_{10}$ CFU/g (Simulation 9; Figure 5A, right). Relatively low temperatures in this simulation led to a slower growth rate. The risk of illness was high beyond Hour 20 and very high beyond Hour 23.

In the DS scenario (Simulation 10; Figure 5B, left), under the same initial conditions, the temperature represented a dockside ice treatment some hours after the harvest of the oysters onboard and transport to the dockside. Here, the ice treatment led to a drastic decrease in temperature; 4 h were necessary for the oysters at high temperature to reach a low temperature (7.2 °C) [16]. Given these temperature conditions, a gradual *V. parahaemolyticus* increase was observed at the beginning of the curve, but the temperatures during cold storage resulted in a slow inactivation process, reaching a maximum of 6.25 $log_{10}$ CFU/g *V. parahaemolyticus* count (Simulation 10; Figure 5B, right). The risk of illness was high beyond Hour 10, but thanks to the DS ice treatment, this risk did not continue increasing and slowly decreased to Hour 50.

Finally, in the OB scenario (Simulation 11; Figure 5C, left), repeating the aforementioned initial conditions, oysters were stored on ice within two hours after harvesting. Therefore, considering that oysters stored on ice reached 7.2 °C in 4 h, beyond Hour 6 of this simulation, the temperature was constant. Under these temperature conditions, a slight increase in the *V. parahaemolyticus* counts was observed up to Hour 4, after which the counts started to gradually decrease, with a final total count of 3.5 $log_{10}$ CFU/g (Simulation 11; Figure 5C, right). The risk of illness in this scenario was low after the drastic inactivation caused by the OB ice treatment.

#### 3.4.2. Simulations 12–14, Summer: Water 25 °C, Air (Max) 32 °C

In the non-ice scenario (Simulation 12; Figure 6A, left), the initial *V. parahaemolyticus* concentration was 1000 CFU/g (3 $log_{10}$ CFU/g), as found by [16] in summer. The postharvest air temperature oscillated between 13 and 32 °C, mirroring day/night temperature fluctuations. Given these varying temperature conditions, a gradual increase in *V. parahaemolyticus* counts was observed with a maximum of around 6.8 $log_{10}$ CFU/g (Simulation 12; Figure 6A, right). The minimum temperatures resulted in slower inactivation. The risk of illness was moderate approximately beyond Hour 25 and high beyond Hour 42, reaching the very high risk of illness at Hour 49.

The DS scenario (Simulation 13; Figure 6B, left) was performed under the same initial conditions previously mentioned. In this scenario, the oysters were initially exposed to temperatures oscillating between 25 and 32 °C. Then, the temperature changed drastically by storing oysters on ice DS for 10 h after harvesting. Then, 4 h were necessary for the oysters on ice to reach a temperature of 7.2 °C, which was maintained to the end of the simulation. The temperatures during ice storage resulted in a halt in the growth, showing a final value of 4.1 $log_{10}$ CFU/g (Simulation 13; Figure 6B, right). The risk of illness increased to low risk by Hour 5 and stayed, with a descending pattern, in that risk zone until the end of the simulation.

Referring to the OB scenario (Simulation 14; Figure 6C, left), under identical initial conditions, after harvesting, it took 2 h for the oysters to be stored on ice. Therefore, after cooling them down, a constant temperature was maintained from Hour 6 to the end of the simulation (Hour 50). A small increase of the *V. parahaemolyticus* counts was observed up to Hour 4, after which the *V. parahaemolyticus* counts started to gradually decrease, with a final total count of 3.1 $log_{10}$ CFU/g (Simulation 14; Figure 6C, right). The risk of illness in this simulation was never higher than low risk.

#### 3.4.3. Simulations 15–17: Winter, Water 15 °C Air (Max) 16 °C

First, alluding to the NI scenario (Simulation 15; Figure 7A, left), the initial *V. parahaemolyticus* concentration value was 10 CFU/g (3 $log_{10}$ CFU/g), as found by [16] in winter. The post-harvest air temperature oscillated between 4 and 16 °C. Given these varying temperature conditions, a gradual decrease in the *V. parahaemolyticus* concentration was observed with 0.85 $log_{10}$ CFU/g at the end of the simulation (Simulation 15; Figure 7A, right). As the temperature did not exceed 16 °C, there was no *V. parahaemolyticus* growth in this scenario, so the risk of illness was very low throughout the simulation.

Continuing with the DS scenario (Simulation 16; Figure 7B, left), a similar trend was observed despite the storage on ice being 10 h after harvesting. A slightly higher *V. parahaemolyticus* inactivation than that observed in the NI scenario can be noticed here, with a final *V. parahaemolyticus* concentration of 0.8 $log_{10}$ CFU/g (Simulation 16; Figure 7B, right). The risk of disease also remained very low throughout the scenario. Finally, with regard to the use of ice OB, the decreasing pattern was also similar to the previous two scenarios; the use of ice after harvesting caused a decrease in the *V. parahaemolyticus* counts, reaching 0.76 $log_{10}$ CFU/g (Simulation 17; Figure 7C, right). The risk of illness also remained at a very low level during the simulation time.

#### 3.4.4. Other Simulations of Interest: Simulations 18–22

In Figure 8, two previously simulated summer scenarios (DS and OB, water 25 °C, air (max) 32 °C) were simulated, but in this case, a hypothetical break in the cold chain occurred. Simulations 18 and 19 (Figure 8A,B, left) reproduced two cold chain break events: the first event described the oysters moved from a market to the consumer (Hour 24, 32 °C), and the second event, at the end of the simulation, refers to the movement of oysters from a market to a consumption site (Hour 50, 29 °C). The model showed an increase in the final concentration of the *V. parahaemolyticus* counts, resulting in *V. parahaemolyticus* concentrations of 4.7 $log_{10}$ CFU/g for the DS scenario (Figure 8A, right) and 3.7 $log_{10}$ CFU/g for the OB scenario (Figure 8B, right). The risk of illness was moderate for the DS scenario and remained low throughout the OB scenario.

Such breaks in cold storage were also tested in winter scenarios (Simulation 20 (DS) and Simulation 21 (OB), water 15 °C, air (max) 18 °C). Although a slight rise in temperature in Hours 24 and 50 was simulated due to those breaks in the cold chain (Figure 9A,B, left), here, the sudden increase in temperature led to a slight increase in *V. parahaemolyticus* that did not compromise the consumer’s health, as the risk of disease remained below the low-risk zone to the end of the simulation (Figure 9A,B, right).

Finally, Figure 10 (DS in summer, air (max) 40 °C) shows how long the use of ice was necessary to reduce the risk of illness to the low-risk zone for gastroenteritis and extremely low for septicemia (Table 3). This simulation was the same as Simulation 10, (Figure 5A, right), but here, applying a more prolonged ice treatment. In this Simulation 22 (Figure 10, left), the oysters were subjected to cooling at 2 °C after the first 10 h of harvesting DS. Referring to [16] and remembering the above, the temperature scenario was constructed as follows: oysters in ice can take an average of 4 h in summer to descend to temperatures equal to 7.2 °C, which is why a temperature equal to 2 °C was reached in Hour 18. This temperature was maintained until the end of the simulation (Hour 300). A gradual inactivation was observed in this simulation, ending with a *V. parahaemolyticus* concentration of 4.5 $log_{10}$ CFU/g, implying a decrease from a high-risk zone to a low-risk zone.

## 4. Discussion

Varying temperatures critically shape the *V. parahaemolyticus* proliferation dynamics of oysters [22,37]. However, empirically testing all possible varying temperature scenarios for a comprehensive study of their effects on *V. parahaemolyticus* proliferation is unachievable. Modeling *V. parahaemolyticus* growth under varying temperatures mirroring ambient temperatures and the effect of ice treatments is crucial to both predict the magnitude of human illnesses and identify the temperature-related dynamics that are most important, particularly in the face of environmental change [12,38].

Our model generalizing from constant to time-varying temperatures was fed by growth and inactivation rate equations, both being temperature-dependent functions. These equations were obtained by means of analyzing the slope of the regression models of the relationship between *V. parahaemolyticus* counts and time under different constant temperatures obtained in previous experiments [21]. The slope for inactivation was identical to that obtained by [21] as the same linear model was applied. The slope for the growth rate (maximum slope) obtained by the non-parametric LOESS regression model was very similar to that obtained by the Baranyi models in [21]. However, the LOESS curve adapted in a more flexible manner to the data and has the potential to extract more information from it than the Baranyi model. That is, the main advantage of using these types of models is the flexibility and the ease of interpretation of the smoothing function [39]. Although further biological/ecological analysis of these non-parametric curves is required, non-parametric curves seem to provide valuable information to study the dynamics of bacterial growth in oysters in detail.

The model verification results conformed to the expectations of mathematical theory, behavior, and population dynamics. Moreover, the model evaluation against real experimental data showed a favorable match with particularly well-documented growth and inactivation dynamics from constant-temperature experiments [21]. The predicted growth rates were also consistent with other experimental studies [25,26]. Once the model has been verified and successfully evaluated, it can be considered for simulating different scenarios with the objective of improving the understanding of the *V. parahaemolyticus*–oyster system to support studies about the public health impact of pathogenic *V. parahaemolyticus* associated with raw oyster consumption. However, the risk assessment results of this model need to be taken with caution until a more robust validation of the model can be performed using experimental *V. parahaemolyticus* growth data under varying temperature scenarios. As far as we know, there are no published data in this regard. In this context, the two main equations of the model (growth/inactivation) performed adequately and showed a satisfactory validation at constant temperatures, so that satisfactory model validation is also expected against *V. parahaemolyticus* growth data under varying temperatures when both equations “work” together. This predictive initiative has the purpose of supporting identifying options for managing diseases caused by the consumption of any type of seafood. In addition, the model has also space to be adapted to and provide insights into other pathogen systems responding to varying temperatures [40].

The model here is able to incorporate varying temperature conditions and day/night temperature oscillations to reproduce their effect on the temporal dynamics of *V. parahaemolyticus*. In this study, the model initial conditions mirrored realistic summer/winter *V. parahaemolyticus* concentrations in oysters [16], with summer *V. parahaemolyticus* counts well above those in winter, as high-temperatures favor *V. parahaemolyticus* proliferation, causing a higher number of bacteria at the time of collection. The model results are discussed here in terms of realistic air temperatures for zones with hot summers and mild winters as in Southern Europe [35] and the Southern U.S. [36]. Increasing ambient temperature in summer favors *V. parahaemolyticus* growth in oysters from 3 $log_{10}$ CFU/g up to 7.5 $log_{10}$ CFU/g, resulting in a very high risk of gastroenteritis after consumption of a serving (12 oysters), particularly in hot summers reaching 40 °C (Simulation 9). The model was developed and evaluated with real data of pathogenic *V. parahaemolyticus* to ultimately predict the risk of illness. However, in a more realistic scenario, pathogenic and nonpathogenic *V. parahaemolyticus* can coexist and TDH+ *Vibrio* spp. growth is slower than that of TDH− *Vibrio* spp. [41,42]. Thus, the application of the model here to a more realistic scenario with TDH- *V. parahaemolyticus* can overestimate the risk of illness. A future version of this model will incorporate this characteristic.

The model also reflects pathogen inactivation due to day/night oscillations, particularly due to ice treatments. Ice treatment is much more effective in limiting the risk of illness due to its strong control on ambient oyster temperatures and, hence, *V. parahaemolyticus* proliferation. Ice is especially effective when applied onboard compared to dockside, where this treatment can exert strong external constraints on initial *V. parahaemolyticus* proliferation following oyster harvest. Although not explicitly considered in our model, immediate onboard icing may reduce the competitive advantage of *V. parahaemolyticus* in oyster-tissue-associated microbial communities, particularly in response to acute changes in temperature [43]. With onboard icing, the gastroenteritis risk decreases to the low-risk zone (Simulation 11), corresponding to an extremely low risk for sepsis (Table 3). For dockside ice treatment, the risk of gastroenteritis after the consumption of a serving of raw oysters is high and the risk of sepsis is very low (Table 3). These results can be extrapolated to milder summers (30 °C) in terms of dynamic patterns (Simulations 12–14) showing a rapid increase of *V. parahaemolyticus* followed by a maximum plateau. However, in this case, both onboard and dockside ice treatments limit the disease risk to the low-risk zone.

The model results were realistic; consuming raw oysters during hot summer months is inherently risky and requires onboard icing, while in mild summers, dockside ice can be an acceptable control measure. The benefit of ice in hot summer scenarios is clear in our model results, but it should be emphasized that late cooling of oysters may not sufficiently inactivate the *V. parahaemolyticus* generated in the first few hours to guarantee safe consumption. These model results were consistent with oyster industry criteria [22] and previous experimental studies [44,45].

For mild winter scenarios (Simulations 15–17), the model showed *V. parahaemolyticus* counts starting from 1 $log_{10}$ CFU/g and remaining consistently below the low-risk zone throughout the tested simulations. The effect of ice on inactivation was slight by Hour 50. Here, the model detected the tiny variations of each scenario; the final concentration of *V. parahaemolyticus* was slightly higher in the non-ice scenario than that observed for the dockside ice treatment scenario and relatively higher when compared to the onboard ice treatment scenario. The model also reproduced the effect of the cold chain breaking in summer (Simulations 18–19), showing an oscillating pattern with *V. parahaemolyticus* growth and inactivation events. This effect was negligible in winter (Simulations 20–21).

A longer preservation time is essential to explore the real extent of cooling oysters down to minimize *V. parahaemolyticus* concentrations. For this, Simulation 22 was performed with initial conditions as in Simulation 10, but running the model for 300 h (almost 13 days) instead of 50 h (2 days). The model reproduced cooling down dockside, decreasing the oyster temperatures to 2 °C. In this long-term simulation, the risk of gastroenteritis decreased from high risk to low risk by Hour 300. Even if the prolonged ice treatment effect seemed to work best for treating oysters for raw consumption, the ice treatment duration needs to be applied with caution. Very long ice treatments risk the marketability of harvested oysters; they may collapse and eventually open, influencing product placement and sales and limiting their consumption [46]. Consequently, conclusions from this simulation result need to be balanced in terms of market quality. In winter, for example, prolonged and strong ice treatments may have little impact on inactivation compared to a non-ice scenario, while the performance of oysters can jeopardize sales.

This obviously cannot be extrapolated to higher-temperature scenarios, where it is necessary to assess the most-appropriate ice treatment timing (onboard or dockside) without posing a risk to human health. In this context, research on new treatments that minimize human health risks is essential. However, achieving this goal will not be an easy task as climatic anomalies are becoming more common, with long summer and winter seasons, thus increasing the seasonal risk of seafood illness. Under such circumstances, it is necessary to consider adjustments in industry practices and regulatory policy, especially for shellfish consumed raw, such as bivalve mollusks [47]. Modeling approaches such as the one presented, verified, and evaluated here can be valuable tools with the necessary adjustments for simulating near-future scenarios under both varying ambient temperatures and the umbrella of climate change [48,49]. In addition, this modeling tool can support predictive studies under irregular and occasional temperature regimes, caused by weather events or the thermoregulatory behaviors of organisms [40]. Overall, this approach will help not only to improve our predictions of how organisms perform in varying temperature environments, but also gain an understanding of how temperature impacts organisms.

## 5. Conclusions

The model for exploring *V. parahaemolyticus* dynamics performed satisfactorily under varying ambient temperatures. The effect of seasons, day/night, and ice-treatment-associated temperature oscillations on the *V. parahaemolyticus* growth patterns was adequately detected and reproduced by the model. Thus, the predictive tool here can serve (i) to improve the understanding of the *V. parahaemolyticus*–oyster system and provide insights into similar marine and terrestrial systems, for which temperature is crucial, and (ii) to support studies about the impact of pathogenic *V. parahaemolyticus* associated with raw oyster consumption on public health, particularly in the face of global change.

## Figures and Tables

**Table 1:** Simulations for model verification and evaluation against experimental data of *V. parahaemolyticus* (*Vp*) growth at constant temperatures from laboratory tests [21].

Simulation	Scenario	Expected Results
Simulation 1	*T* = 18.4 °C, *Vp* = 3.4 $log_{10}$ CFU/g	Logistic (extension) 3.4 to 5.5 $log_{10}$ CFU/g
Simulation 2	*T* = 20 °C, *Vp* = 3.4 $log_{10}$ CFU/g	Logistic (extension) 3.4 to 5.5 $log_{10}$ CFU/g
Simulation 3	*T* = 25.7 °C, *Vp* = 3.4 $log_{10}$ CFU/g	Logistic (extension) 3.4 to 7.5 $log_{10}$ CFU/g
Simulation 4	*T* = 30.4 °C, *Vp* = 3.4 $log_{10}$ CFU/g	Logistic (extension) 3.4 to 6.75 $log_{10}$ CFU/g
Simulation 5	*T* = 3.6 °C, *Vp* = 5.8 $log_{10}$ CFU/g	Linear 5.8 to 3.0 $log_{10}$ CFU/g
Simulation 6	*T* = 6.2 °C, *Vp* = 5.5 $log_{10}$ CFU/g	Linear 5.5 to 4.0 $log_{10}$ CFU/g
Simulation 7	*T* = 9.6 °C, *Vp* = 5.1 $log_{10}$ CFU/g	Linear 5.1 to 3.0 $log_{10}$ CFU/g
Simulation 8	*T* = 12.6 °C, *Vp* = 5.3 $log_{10}$ CFU/g	Linear 5.3 to 4.0 $log_{10}$ CFU/g

**Table 2:** Realistic scenarios for exploring *V. parahaemolyticus* (*Vp*) dynamics in oysters under varying temperature. Ice treatment types as Non-Iced (NI), Dockside (DS) ice treatment, Onboard (OB) ice treatment, Breaking Cold Chain (BCC), season (summer, winter), Water temperature at harvest (W), maximum Air temperature (A), and initial *Vp* number per oyster gram are defined for each scenario.

Simulation	Scenario
Simulation 9	NI, Summer, W = 30 °C, A = 40 °C, *Vp* = 3 $log_{10}$ CFU/g
Simulation 10	DS, Summer, W = 30 °C, A = 40 °C, *Vp* = 3 $log_{10}$ CFU/g
Simulation 11	OB, Summer, W = 30 °C, A = 40 °C, *Vp* = 3 $log_{10}$ CFU/g
Simulation 12	NI, Summer, W = 25 °C, A = 32 °C, *Vp* = 3 $log_{10}$ CFU/g
Simulation 13	DS, Summer, W = 25 °C, A = 32 °C, *Vp* = 3 $log_{10}$ CFU/g
Simulation 14	OB, Summer, W = 25 °C, A = 32 °C, *Vp* = 3 $log_{10}$ CFU/g
Simulation 15	NI, Winter, W = 15 °C, A = 16 °C, *Vp* = 1 $log_{10}$ CFU/g
Simulation 16	DS, Winter, W = 15 °C, A = 16 °C, *Vp* = 1 $log_{10}$ CFU/g
Simulation 17	OB, Winter, W = 15 °C, A = 16 °C, *Vp* = 1 $log_{10}$ CFU/g
Simulation 18	DS, BCC Summer, W = 30 °C, A = 32 °C, *Vp* = 3 $log_{10}$ CFU/g
Simulation 19	OB, BCC, Summer, W = 30 °C, A = 32 °C, *Vp* = 3 $log_{10}$ CFU/g
Simulation 20	DS, BCC, Winter, W = 15 °C, A = 18 °C, *Vp* = 1 $log_{10}$ CFU/g
Simulation 21	OB, BCC, Winter, W = 15 °C, A = 18 °C, *Vp* = 1 $log_{10}$ CFU/g
Simulation 22	DS, Summer, W = 25 °C, A = 40 °C, *Vp* = 3 $log_{10}$ CFU/g

**Table 3:** Probability and Risk of Gastroenteritis ((P(G) and R(G), respectively) and Septicemia (P(S) and R(S), respectively) as a function of *V. parahaemolyticus* dose per oyster serving (12 oysters) and per oyster gram in a serving. Adapted and estimated from results of the Beta-Poisson dose–response model by [22].

CFU per Serving	Log CFU/g	P (G)	Risk (G)	P (S)	Risk (S)
$10^{4}$	1.72	$1.0\;\times \;10^{-4}$	Extremely low	$2.5\;\times \;10^{-7}$	Extremely low
$10^{5}$	2.72	$1.0\;\times \;10^{-3}$	Very low	$2.5\;\times \;10^{-6}$	Extremely low
$10^{6}$	3.72	$1.0\;\times \;10^{-2}$	Low	$2.5\;\times \;10^{-5}$	Extremely low
$10^{7}$	4.72	$1.0\;\times \;10^{-1}$	Moderate	$2.5\;\times \;10^{-4}$	Extremely low
$10^{8}$	5.72	$5.0\;\times \;10^{-1}$	High	$1.3\;\times \;10^{-3}$	Very low
$10^{9}$	6.72	$9.0\;\times \;10^{-1}$	Very high	$2.3\;\times \;10^{-3}$	Very low
$10^{10}$	7.72	$9.6\;\times \;10^{-1}$	Extremely high	$2.4\;\times \;10^{-3}$	Very low
$10^{11}$	8.72	$9.9\;\times \;10^{-1}$	Extremely high	$2.5\;\times \;10^{-3}$	Very low

## Data Availability

The original contributions generated for the study are included in the article; further inquiries about the data can be directed to the corresponding author. The code of the model is available at https://rb.gy/svwchb, Matlab File Exchange Repository (accessed on 10 February 2023).
